# The Impact of Upward Social Comparison on Smartphone Addiction Among Adolescents: The Moderating Role of Football Participation

**DOI:** 10.3390/bs16030346

**Published:** 2026-02-28

**Authors:** Anzu Li, Huarui Huang, Yi Zheng, Nian Li, Yizhou Shui

**Affiliations:** 1 School of Physical Education, Shaanxi Normal University, Xi’an 710119, China; llianzu@snnu.edu.cn (A.L.); huanghuarui@snnu.edu.cn (H.H.); zhengyi@snnu.edu.cn (Y.Z.); 2 School of Psychology, Shaanxi Normal University, Xi’an 710062, China

**Keywords:** adolescent, football participation, smartphone addiction, sports participation, upward social comparison

## Abstract

Against the background of widespread digital technology use and the increasing prevalence of smartphone addiction among adolescents, upward social comparison has been identified as an important trigger of addictive behaviors. However, the moderating role of sports participation and potential differences across types of sports remain unclear. This cross-sectional study aimed to examine the effect of upward social comparison on smartphone addiction among adolescents and to test the moderating role of football participation. A questionnaire survey was conducted among 2451 primary and secondary school students from 162 schools across 13 provinces in China. The survey included the Upward Social Comparison Scale, the Smartphone Addiction Scale—Short Version, and the Sports Participation Scale. SPSS 27.0 was used to perform descriptive statistics and correlation analyses. Moderation analyses and multi-group comparisons were conducted using PROCESS 4.2. The results showed that upward social comparison positively predicted smartphone addiction among adolescents. Sports participation significantly buffered the association between upward social comparison and smartphone addiction (*β* = −0.055, *p* < 0.001). Football participation showed a significant moderating effect (*β* = −0.062, *p* < 0.05). Higher levels of football participation were associated with a stronger buffering effect. In contrast, individual sports did not show a significant moderating effect (*β* = −0.029, *p* = 0.394). These findings suggest that upward social comparison is a risk factor for smartphone addiction among adolescents. Football participation may effectively reduce this risk, but individual sports did not exhibit a comparable moderating effect. This study provides empirical evidence to support the development of targeted intervention strategies for adolescent smartphone addiction.

## 1. Introduction

In the 21st century, the deep penetration of digital technology, particularly the proliferation of mobile internet, has driven a rapid expansion in the number of mobile phone users. Today, mobile phones are owned by 90% of the global population (Zeng et al., 2022). Smartphones provide important support for teenagers’ education and social interaction. During the pandemic, they allowed students to attend classes, submit work, access materials, and stay connected socially, thereby lowering the health risks of physical contact (Wang et al., 2023). However, this high level of reliance has resulted in problematic smartphone addiction. Smartphone addiction is mainly characterized by cognitive conflict, loss of control, emotional dysregulation, and compulsive use. These characteristics can lead to serious psychological, social, and physical consequences (Kwon et al., 2013). Adolescents are particularly vulnerable because their brains are still developing. An immature prefrontal cortex weakens self-control (Arain et al., 2013). At the same time, a sensitive dopamine system increases reward-seeking behavior (Reichelt, 2016). This combination of physiological and psychological vulnerability makes adolescents a high-risk group for smartphone addiction.

When examining the causes of smartphone addiction, upward social comparison is recognized as a significant contributing factor (He et al., 2020). Upward social comparison specifically describes the habit of comparing oneself to others perceived as more successful or advantaged (Collins, 1996). From the perspective of the triadic reciprocal determinism of social cognitive theory, adolescent smartphone addiction results from the dynamic interaction among environmental, cognitive, and behavioral factors (Bandura, 1986). The pervasive display of perfect lives on social media provides adolescents with frequent opportunities for upward social comparison. Through observational learning, they are exposed to a large amount of filtered and polished information in this environment (Qi et al., 2024). Due to their still-developing cognitive systems and limited critical appraisal abilities, adolescents are more likely to form the belief that others are better than themselves. This cognitive bias often leads to negative self-appraisal. Negative self-appraisal is associated with adverse emotional states (Samra et al., 2022). Smartphones can provide immediate emotional gratification. To relieve these negative emotions, adolescents may increasingly rely on smartphone use, which can eventually lead to smartphone addiction (S. Jiang et al., 2024).

Sports participation serves as an effective intervention to mitigate smartphone addiction stemming from upward social comparison. According to social cognitive theory, environmental factors shape behavior by influencing cognition, and individuals use cognitive regulation to reinterpret their environment, leading to different behavioral choices (Bandura, 1986). Within this framework, upward social comparison serves as an external environmental input. It reduces individuals’ self-efficacy and leads to negative self-perceptions (Egele et al., 2025). And sports participation may act as a buffer by regulating these cognitive processes (He et al., 2020). Specifically, as an effective source of mastery experiences, exercise can optimize an individual’s cognitive processes (Kleppang et al., 2023; Urdan & Pajares, 2006). This cognitive optimization weakens the negative impact of upward social comparison (Tian et al., 2024). As a result, it reduces the risks associated with smartphone usage behavior (Servidio et al., 2021).

Notably, the effects of exercise interventions may be due to differences in social structure. Compared with individual sports, team sports offer stronger social support and closer social connections (Wan et al., 2025). They also provide a richer setting for cognitive and behavioral adjustment, as emphasized by social cognitive theory (Wei et al., 2025). In such environments, individuals’ psychological resilience and self-identity are strengthened (M. Li et al., 2025). This enhancement enables them to better withstand the negative impacts of upward social comparisons (X. Jiang et al., 2025). Ultimately, this makes it more likely to reduce the risk of smartphone addiction arising from negative emotions or unmet psychological needs (Luo et al., 2025). Among team sports, football (which refers to soccer rather than U.S. football in this study) stands out for its intensive cooperation, rich social interaction, and popularity among adolescents (Huang et al., 2025b). Therefore, football is often used as an important intervention to address internet addiction among adolescents (Shan et al., 2025). Football can enhance their cognitive functions, such as attention and inhibitory control (Mao et al., 2024). It also strengthens psychological resilience through team support and shared achievement (Zheng et al., 2025). Upward social comparison often triggers negative cognitive biases and emotional exhaustion (McComb et al., 2023). Football improves cognitive functions and self-control, which help individuals regulate their emotions more effectively (Xie et al., 2021). This emotional regulation can buffer adverse psychological reactions (Daniel et al., 2020). In digital contexts, such negative emotions often lead to problematic smartphone use as a coping strategy (Giansanti, 2025). Thus, by building these protective capacities, football maybe lower the addiction risk stemming from upward social comparison.

Previous studies have seldom distinguished between different types of sports when examining the links between upward social comparison and smartphone addiction. To address this gap, this study proposes a moderation model, and three hypotheses (H) are proposed:

**H1.**  *Adolescents who engage in higher levels of upward social comparison are likely to exhibit more severe symptoms of smartphone addiction.*

**Figure 1:**
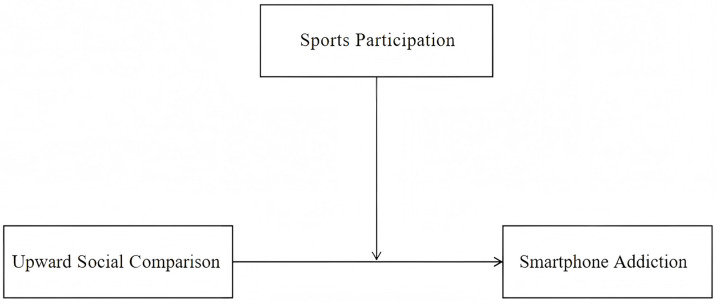
The moderating effect of sports participation on the relationship between upward.

**H2.**  *Higher levels of sports participation can predict lower symptoms of smartphone addiction and buffer the negative impact of upward social comparison. (Figure 1).*

**Figure 2:**
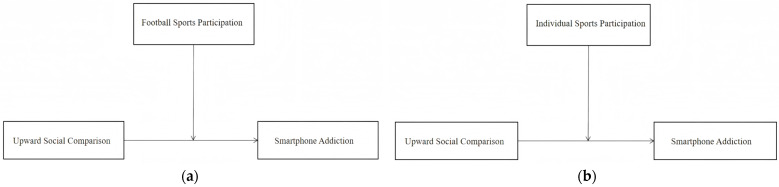
(**a**) The moderating effect of football participation on the relationship between upward social comparison and smartphone addiction; (**b**) the moderating effect of individual sports participation on the relationship between upward social comparison and smartphone addiction.

**H3.**  *This buffering effect will be stronger among football players than among individual sports participants. (Figure 2).*

## 2. Materials and Methods

### 2.1. Participants

After we obtained written informed consent from all participants and their legal guardians, we distributed the questionnaires uniformly in primary and secondary school classes at the participating schools. In total, 2451 adolescents from primary school to high school participated in the study. Of these, 2435 completed the questionnaire with no missing data. This yielded a response rate of 99.4%. The mean age was 12.36 years (*SD* = 3.42). Participants were recruited from 162 schools across 13 provinces. These schools covered all seven major geographic regions of China. Of the total sample, 1510 participants (62%) were involved in football. They had received formal football training. They were also required to take part in structured football activities at least three times per week. The remaining 925 participants (38%) engaged in individual sports, such as athletics, swimming, and other comparable individual-based physical activities. This group included students who had not received any formal football training.

### 2.2. Procedure

Ethical approval for this study was obtained from the Ethics Committee of Shaanxi Normal University (Approval No. 202616008). Permission to conduct the survey was granted by the participating schools. Prior to data collection, written informed consent was obtained from students’ legal guardians, and assent was obtained from the students themselves. All participants were informed about the purpose of the study, the research procedures, and data protection measures. Participation was entirely voluntary. Data were collected anonymously, and all information was kept confidential and used exclusively for research purposes.

### 2.3. Measurement

#### 2.3.1. General Information

The general information questionnaire collected demographic data, including gender (1 = male, 2 = female), level of school (1 = primary school, 2 = junior high school, 3 = senior high school), and sport-type (1 = football, 2 = individual sport). Prior research shows that demographic factors like gender and school level can influence the relationship between football and related psychological outcomes (Huang et al., 2025a). Accordingly, we included gender and school level as covariates in our analyses to control for their potential effects.

#### 2.3.2. Upward Social Comparison Subscale (USC)

This study used the Chinese version of the Upward Social Comparison subscale, which was adapted by Bai Xuejun from the original scale developed by Gibbons and Buunk (Bai et al., 2013; Gibbons & Buunk, 1999). The Chinese version has demonstrated good reliability and validity (Lian et al., 2017). The subscale consists of 6 items rated on a five-point Likert scale (1 = strongly disagree, 5 = strongly agree). One example of an item is as follows: “When using social media, I often like to compare myself with those who are better off than me.” In this study, the Cronbach’s α was 0.895.

#### 2.3.3. Smartphone Addiction (SAS-SV)

Smartphone addiction was measured using the Smartphone Addiction Scale—Short Version (SAS-SV), developed by Kwon (Kwon et al., 2013). The scale was translated into Chinese by Xiang and has been shown to have good validity and reliability (Xiang et al., 2019). The SAS-SV consists of 10 items rated on a 6-point Likert scale (1 = strongly disagree, 6 = strongly agree). One example of an item is as follows: “I feel impatient and fretful when I am not holding my smartphone.” In this study, the Cronbach’s α was 0.933.

#### 2.3.4. Athlete Participation Scale (APS)

Sports participation was measured using the Athlete Participation Scale. originally developed by Agbuga et al. (2016). The scale was translated into Chinese by Zhang (2023). Previous research has demonstrated that the Chinese version of the scale exhibits satisfactory reliability and validity (Shan et al., 2025). The entire scale consists of 13 items rated on a 5-point Likert scale (1 = strongly disagree, 5 = strongly agree). One example of an item is as follows: “I continuously monitor my athletic performance during training classes.” In this study, the Cronbach’s α was 0.987.

#### 2.3.5. Data Analysis

All statistical analyses were conducted using SPSS 27.0. First, the data were screened for missing values. No missing data were detected. Therefore, all valid cases were retained for subsequent analyses. Second, descriptive statistics and bivariate correlations were computed for all study variables. Third, Model 1 (simple moderation model) of Hayes’ PROCESS macro was used to examine the hypothesized moderating effect (Hayes, 2017). Gender and school level were included as covariates in all subsequent models. This model tested whether sports participation moderated the association between upward social comparison and smartphone addiction. Finally, a multi-group moderation analysis was performed to examine differences across types of sports activities.

## 3. Results

### 3.1. Basic Characteristics

The initial sample comprised 2451 adolescents from primary to high school. Of these, 2435 provided complete and valid responses. The validity rate was 99.4%. Among the 2435 valid participants, 1809 (74.3%) were male and 626 (25.7%) were female. A total of 721 participants (29.6%) were aged 6–10 years, 1367 (56.1%) were aged 11–15 years, and 347 (14.3%) were aged 16–20 years. In addition, 1352 (55.5%) were in primary school, 666 (27.4%) were in junior high school, and 417 (17.1%) were in senior high school. Furthermore, 1510 participants (62.0%) participated in football and 925 (38.0%) participated in individual sports. Detailed sociodemographic characteristics are presented in Table 1.

### 3.2. Common Method Bias Test

The Harman single-factor test shows that there are 4 factors with eigenvalues greater than 1. The first factor explains 40.09% of the variance, which does not exceed the critical standard of 50% (Podsakoff & Organ, 1986), indicating that common method bias was not a serious concern in this study.

### 3.3. Descriptive Statistics and Correlation Analysis

The descriptive statistics of each variable are shown in Table 2, depicting the Pearson correlation analysis for the total sample (*n* = 2435). Although the correlation coefficients between all variables are not particularly strong, they are all highly significant. Specifically, sports participation was negatively correlated with smartphone addiction (*p* < 0.001) and upward social comparison (*p* < 0.001), while upward social comparison was positively correlated with smartphone addiction (*p* < 0.001).

### 3.4. Moderating Effect

Moderation analyses were conducted using Hayes’ PROCESS Model 1 (Hayes, 2017). Upward social comparison was specified as the independent variable (X), sports participation as the moderator (W), and smartphone addiction as the dependent variable (Y). The unstandardized coefficients are presented in the Supplementary Materials, while the standardized results are shown in Table 3. Upward social comparison positively predicted smartphone addiction (*β* = 0.122, *p* < 0.001), and sports participation negatively predicted smartphone addiction (*β* = −0.119, *p* < 0.001); moreover, the interaction between upward social comparison and sports participation was significant (*β* = −0.055, *p* < 0.001), indicating that sports participation significantly attenuated the positive association between upward social comparison and smartphone addiction. Notably, although the interaction effect achieved statistical significance, the actual effect size reflected by the *β* value was relatively weak.

**Figure 3:**
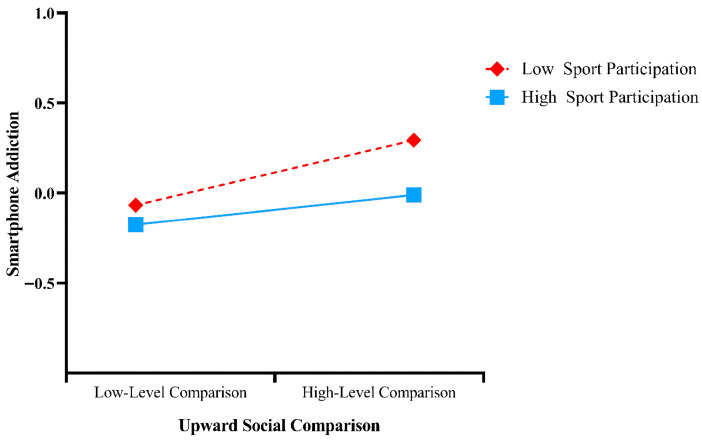
Figure of the moderating effect of sports participation.

In addition, a simple slope analysis (Figure 3) (Aiken et al., 1991) indicated that the buffering effect of sports participation on the relationship between upward social comparison and smartphone addiction was stronger at a high level of sports participation (*M* + 1 *SD*; *simple slope* = 0.081, *t* = 3.431, *p* < 0.001) than at a low level of sports participation (*M* − 1 *SD*; *simple slope* = 0.177, *t* = 6.09, *p* < 0.001).

Subsequently, the study employed upward social comparison (X) as the independent variable, football participation (W) as the moderating variable, and smartphone addiction (Y) as the dependent variable. The unstandardized coefficients are presented in the Supplementary Materials, while the standardized results are shown in Table 4. Upward social comparison predicts smartphone addiction (*β* = 0.160, *p* < 0.001), football participation is negatively associated with smartphone addiction (*β* = −0.065, *p* = 0.013), and key interactive item participation has a negative predictive effect on the moderating role of upward social comparison and smartphone addiction (*β* = −0.062, *p* = 0.010), indicating that football participation significantly attenuated the positive association between upward social comparison and smartphone addiction.

**Figure 4:**
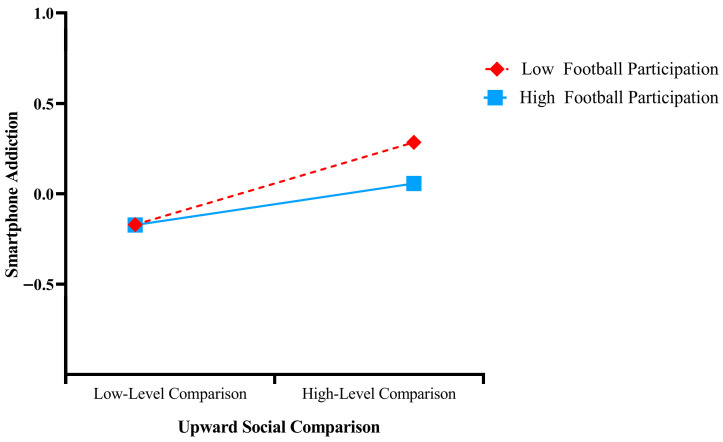
Figure of the moderating effect of football participation.

In addition, a simple slope analysis (Figure 4) (Aiken et al., 1991) indicated that the buffering effect of football participation on the relationship between upward social comparison and smartphone addiction was stronger at a high level of football participation (*M* + 1 *SD*; *simple slope* = 0.112, *t* = 3.674, *p* < 0.001) than at a low level of football participation (*M* − 1 *SD*; *simple slope* = 0.222, *t* = 6.114, *p* < 0.001).

Finally, the study employed upward social comparison (X) as the independent variable, individual sports participation (W) as the moderating variable, and smartphone addiction (Y) as the dependent variable. The unstandardized coefficients are presented in the Supplementary Materials, while the standardized results are shown in Table 5. The moderating effect of individual sports participation on upward social comparison and smartphone addiction was not significant. (*β* = −0.029, *p* = 0.394).

## 4. Discussion

Grounded in social cognitive theory, this study examined the effect of upward social comparison on smartphone addiction among adolescents, as well as the moderating role of sports participation. The study further compared the moderating effects of football and individual sports. The results showed that upward social comparison significantly and positively predicted smartphone addiction. Sports participation weakened this association. Football significantly attenuated the effect of upward social comparison on smartphone addiction, whereas individual sports did not show a significant moderating effect. Notably, although some effect sizes were relatively small in the present study, these results still reached statistical significance, which is a common phenomenon in social science research with large sample sizes (Matz et al., 2017).

Consistent with H1, our findings confirm that upward social comparison positively predicted smartphone addiction symptoms. This result is not only consistent with previous studies (He et al., 2020), but also aligns with social cognitive theory, which posits that an individual’s social environment significantly shapes their cognition and behavior (Bandura, 1986). As a common social psychological process, upward social comparison alters individuals’ emotional states. It also influences self-evaluation and sense of worth through cognitive processing (Muller & Fayant, 2010). Because the prefrontal cortex in adolescents has not yet fully developed, their executive functions and metacognitive abilities remain relatively limited (Meredith & Silvers, 2024). When exposed to upward social comparison, adolescents are therefore more likely to develop negative self-perceptions and emotional distress (Crone & Dahl, 2012). In such contexts, smartphones serve as a convenient compensatory tool to alleviate discomfort caused by cognitive imbalance and emotional depletion (Elhai et al., 2017). With increased frequency and intensity of upward social comparisons, dependence on smartphones is strengthened, thereby elevating the subsequent risk of addictive use (Fu et al., 2020).

Consistent with H2, this study found that higher levels of sports participation predicted lower levels of smartphone addiction symptoms and buffered the negative impact of upward social comparison. These findings are consistent with the core propositions of social cognitive theory. According to this theory, self-efficacy is a central element in individual cognitive regulation (Bandura, 1986). Upward social comparison is an external social information, but its impact depends on how individuals understand and evaluate this comparison (Muller & Fayant, 2010). Adolescents who engage in regular sports develop stronger self-efficacy and a greater sense of control through overcoming difficulties and learning new skills (X. Li et al., 2024). These positive beliefs help them maintain constructive thinking and reduce negative emotions when facing upward social comparisons (Y. Li, 2019). In addition, regular physical activity improves mood and mental health through the release of dopamine and endorphins (Holland et al., 2024). When adolescents use exercise to regulate their emotions, they are more likely to relieve stress through this healthy approach rather than relying on smartphones for comfort (Wu et al., 2024). From the perspective of behavioral habits, regular exercise requires time investment and sustained attention. This requirement reduces the amount of time adolescents spend using smartphone (Ye et al., 2025). By maintaining a more structured daily routine, adolescents are less likely to engage in upward social comparison on social media (Irmer & Schmiedek, 2023; McComb et al., 2023). Regular exercise also enhances self-discipline and attention, helping adolescents better regulate their daily behaviors (Zhao et al., 2025). Such enhanced self-control further facilitates effective emotion regulation, thereby reducing emotionally driven smartphone use (Ding et al., 2022).

This study found that sports participation was generally associated with lower levels of smartphone addiction. However, the strength of this association varied by sport type. No significant moderating effect was observed for individual sports, whereas a clear moderating effect was found for team sports such as football. Specifically, higher levels of football participation weakened the positive association between upward social comparison and smartphone addiction. These findings support H3.

According to the triadic reciprocal model of social cognitive theory, the unique social context of football may strengthen both cognitive and social resources. This process helps buffer the impact of upward social comparison on smartphone addiction. The dynamic and unpredictable nature of football requires players to continuously integrate information from teammates, opponents, and the ball. This demand enhances key cognitive functions, including attentional control and response inhibition (Pruna & Bahdur, 2016). Strengthening these cognitive abilities can help adolescents view the idealized images presented on social media more rationally. This reduces anxiety caused by upward social comparison (Chen et al., 2023), and reduces their propensity to use phones as a form of escape (Elhai et al., 2019). From a physiological development perspective, football relies mainly on lower-limb movements to execute technical actions. These movements occur in body regions far from the brain. They may better activate cerebellar–prefrontal neural circuits linked to executive function. This activation may promote the maturation of attentional control and self-regulation. It may also help adolescents control impulses and stabilize emotions when facing upward social comparison. As a result, adolescents may be less likely to use smartphones to relieve negative emotions (Tauste-Garcia et al., 2025). This may, in turn, buffer the effect of upward social comparison on smartphone addiction. From a cognitive development perspective, football is played in a large, open, and rapidly changing environment. It requires players to observe, anticipate, decide, and adapt to changes on the field. This process may improve cognitive control, sustained attention, and information-processing speed (Zhong et al., 2025). It may also help adolescents process upward social comparison information more rationally. In turn, it may reduce impulsive and emotion-driven smartphone use. This may mitigate the negative effect of upward social comparison on smartphone addiction. In addition, positional roles in football are both clearly defined and flexible. This structure requires players to understand their individual responsibilities and develop a stable sense of self-identity (Popovych et al., 2021; Yan et al., 2025). At the same time, frequent role switching helps players adapt to different tactical situations and improve their teamwork and decision-making skills (Machado et al., 2019). This combination may make social comparisons feel less threatening. It also encourages individuals to view perceived gaps as opportunities for learning and improvement. As a result, adolescents may experience more positive emotions and stronger motivation to improve (Van de Ven, 2017; Yuviler-Gavish et al., 2024). This process further reduces compensatory smartphone use (Shao et al., 2024). Due to the low scores and high volatility in football (Scarf et al., 2022), athletes regularly face failure. This process enhances their psychological resilience and uncertainty tolerance (Saward et al., 2023). This helps individuals maintain a balanced mindset during upward social comparison, thereby avoiding frustration-related distress (Y. Li & Zheng, 2025). A more stable emotional state further decreases avoidance-oriented smartphone use driven by negative emotions (Türk-Kurtça & Kocatürk, 2025).

Different from football participation, individual sports participation was associated with lower levels of smartphone addiction, but did not significantly weaken the relationship between upward social comparison and smartphone addiction. This may be because individual sports do not provide sufficient psychological support to reduce the effect of upward social comparison on smartphone addiction. First, existing studies suggest that various forms of sports can help reduce smartphone addiction (Liu et al., 2019). However, individual sports offer weaker social support than team sports. As a result, it is more difficult for athletes to develop stable self-esteem and effective emotion regulation (Portela-Pino et al., 2024; Reardon & Hitchcock, 2024). Without the social support provided by a team, frustration arising from upward social comparison may lead individuals to experience self-doubt and psychological threat. Individuals with this perception may use smartphones to avoid negative emotions (Pikó et al., 2024). Secondly, given the more limited emotional feedback in individual sports, athletes’ motivation often depends more on the internal satisfaction of self-challenge (Pradeep & Ajeesh, 2013). Although this internal motivation can drive effort, it can be unstable. Individual athletes often face setbacks or failures without immediate emotional support from peers. They must handle the resulting pressure alone, which can increase mood swings and undermine motivation (Simons & Bird, 2023). As a result, athletes may be more likely to use smartphones to cope with negative emotions and seek comfort (Squires et al., 2021). Finally, individual sports focus on personal goals and self-improvement (Tomczak et al., 2024). When they compare themselves to better-performing others, they may blame themselves for not doing well. This self-blame can lead to negative emotions. Compared to team sports, individual sports involve fewer opportunities for real-time interpersonal feedback and shared emotional regulation (Dias et al., 2010). As a result, adolescents have limited help to ease upward social comparison-related stress. In addition, individual sports make it harder to develop team belonging and shared goals (Riedl et al., 2025). Without a strong sense of belonging, adolescents may be more likely to question their self-worth when facing setbacks. They also cannot rely on group understanding or collective support (White-Gosselin et al., 2023). Therefore, although individual sports can reduce smartphone addiction, they do not exert a significant moderating effect on the relationship between upward social comparison and smartphone addiction.

Based on social cognitive theory, this study explains how upward social comparison contributes to smartphone addiction among adolescents. It also confirms the moderating role of sports participation and highlights the unique buffering effect of football. The findings not only extend social cognitive theory to digital contexts but also provide a theoretical perspective on adolescent addiction. The results indicate that sports participation, particularly team sports such as football, can weaken the effect of upward social comparison on smartphone addiction. This effect is achieved through enhanced self-efficacy and increased social support (Kleppang et al., 2023; Panza et al., 2022). From a practical perspective, the findings suggest that schools should encourage participation in team sports such as football. This approach may help students develop team spirit and psychological resilience, thereby promoting healthier patterns of social comparison (Huang et al., 2025a). Overall, this study offers a sports-based strategy for addressing adolescent smartphone addiction and has implications for both theory and practice.

This study has several limitations. First, it employed a cross-sectional design, which can only reveal correlations among variables rather than establish causal relationships (Maxwell & Cole, 2007). Future studies could use longitudinal cross-lagged panel model to further test the causal mechanisms. Secondly, smartphone addiction is a complex psychosocial phenomenon affected by multiple factors. It is influenced not only by sports participation but also by family environment, parent–child relationship, peer influence, socioeconomic status, and other related factors (Busch & McCarthy, 2021). However, the present study only focused on the comparison of moderating effects between different types of sports, and these important influencing factors were not included. This may also explain why the correlation and the moderating effect identified in this study are both relatively weak. Future research should take these factors into account to establish a more comprehensive model. Third, although self-report measures are valuable for assessing subjective experiences, they are susceptible to common method bias (Jahedi & Méndez, 2014; Podsakoff et al., 2003). To address this limitation, future research should incorporate diverse data sources or objective measures. Such approaches would help reduce potential bias and allow for a more comprehensive assessment of smartphone addiction among adolescents.

## 5. Conclusions

This study demonstrates that upward social comparison predicts smartphone addiction among adolescents. Sports participation moderated this relationship overall. However, a significant moderating effect was observed only for football participation, whereas individual sports showed no such effect. These findings extend the application of social cognitive theory in the sports domain and provide a practical basis for developing targeted football-based intervention strategies. Such strategies can enhance adolescents’ cognitive control, emotional regulation, psychological resilience, and social support, thereby alleviating the negative impacts of upward social comparison. This can further help reduce smartphone addiction, promote healthier digital habits, and support the overall mental and physical development of adolescents. Accordingly, these results carry important implications for both theory and practice.

## Figures and Tables

**Table 1:** Sociodemographic characteristics (*n* = 2435).

Variable	Frequency	Percentage
**Gender**		
Male	1809	74.3%
Female	626	25.7%
**Age**		
6–10 years	721	29.6%
11–15 years	1367	56.1%
16–20 years	347	14.3%
**Level of school**		
Primary school	1352	55.5%
Junior high school	666	27.4%
Senior high school	417	17.1%
**Sports**		
Football	1510	62.0%
Individual	925	38.0%

**Table 2:** Descriptive statistics and correlation analysis (*n* = 2435).

Variables	*M*	*SD*	1	2	3
Sports Participation	4.081	1.219	1		
Smartphone Addiction	2.402	1.243	−0.141 ***	1	
Upward Social Comparison	3.342	1.057	−0.079 ***	0.130 ***	1

Note: The mean is denoted as *M*, and the standard deviation is denoted as *SD*. ***, *p* < 0.001.

**Table 3:** Sports participation moderation effect test (*n* = 2435).

Variables	*β*	*SE*	*t*	LLCI	ULCI
Upward Social Comparison (X)	0.122 ***	0.020	6.101	0.828	0.161
Sports Participation (W)	−0.119 ***	0.021	−5.805	−0.159	−0.079
X × W	−0.055 ***	0.019	−2.841	−0.092	−0.017
Constant	0.037	0.073	0.508	−0.107	0.181

Note: All coefficients are standardized. ***, *p* < 0.001.

**Table 4:** Football participation moderation effect test (*n* = 1510).

Variables	*β*	*SE*	*t*	LLCI	ULCI
Upward Social Comparison (X)	0.160 ***	0.025	6.286	0.110	0.210
Sports Participation (W)	−0.065 *	0.026	−2.477	−0.116	−0.013
X × W	−0.062 *	0.024	−2.569	−0.110	−0.145
Constant	−0.064	0.093	−0.693	−0.246	0.117

Note: All coefficients are standardized. *, *p* < 0.05; ***, *p* < 0.001.

**Table 5:** Test of moderating effect of individual sports participation (*n* = 925).

Variables	*β*	*SE*	*t*	LLCI	ULCI
Upward Social Comparison (X)	0.066 *	0.033	2.015	0.002	0.130
Sports Participation (W)	−0.217 ***	0.032	−6.758	−0.280	−0.154
X × W	−0.029	0.034	−0.853	−0.096	0.038
Constant	0.207	0.120	1.724	−0.029	0.444

Note: All coefficients are standardized. *, *p* < 0.05; ***, *p* < 0.001.

## Data Availability

Data are available from the corresponding author upon reasonable request.

## Supplementary Materials

The following supporting information can be downloaded at: https://www.mdpi.com/article/10.3390/bs16030346/s1.
