# Supplementary material for: The Impact of Upward Social Comparison on Smartphone Addiction Among Adolescents: The Moderating Role of Football Participation
Source: Behav Sci (Basel). 2026 Feb 28;16(3):346. doi: 10.3390/bs16030346 (PMC13024103; doi:10.3390/bs16030346)
Supplement: Supplementary file 1 [file behavsci-16-00346-s001.zip › behavsci-4141739-supplementary.pdf]

Supplementary Materials

Moderation analyses were conducted using Hayes' PROCESS Model 1 (Hayes, 2017). Upward social comparison was specified as the independent variable (X), sports participation as the moderator (W), and smartphone addiction as the dependent variable (Y). The results are presented in Table 3. Upward social comparison positively predicted smartphone addiction (b = 0.239, p < 0.001), sports participation negatively predicted smartphone addiction (b = −0.093, p < 0.001), moreover, the interaction between upward social comparison and sports participation was significant (b = −0.007, p < 0.001), indicating that sports participation significantly attenuated the positive association between upward social comparison and smartphone addiction.

Sports Participation Moderation Effect Test (n = 2435).

Variables	Coefficient	*SE*	*t*	LLCI	ULCI
Upward Social Comparison (X)	0.239 ***	0.039	6.101	0.162	0.316
Sports Participation (W)	−0.093 ***	0.016	−5.805	−0.125	−0.062
X × W	−0.007 ***	0.002	−2.841	−0.011	−0.002
Constant	24.482 ***	0.911	26.881	22.696	26.268

Note: *, p < 0.05; **, p < 0.01; ***, p < 0.001.

Subsequently, the study employed upward social comparison (X) as independent variable, football participation (W) as moderating variable, and smartphone addiction (Y) as dependent variable, the results are shown in Table 4. Upward social comparison predicts smartphone addiction (b = 0.324, p < 0.001), football participation is negatively associated with smartphone addiction (b = −0.047, p = 0.013), key interactive item participation has a negative predictive effect on the moderating role of upward social comparison and smartphone addiction (b = −0.007, p = 0.010), indicating that football participation significantly attenuated the positive association between upward social comparison and smartphone addiction.

Football Participation Moderation Effect Test (n = 1510).

Variables	Coefficient	*SE*	*t*	LLCI	ULCI
Upward Social Comparison (X)	0.324 ***	0.052	6.286	0.223	0.425
Sports Participation (W)	−0.047 *	0.019	−2.477	−0.083	−0.010
X × W	−0.007 *	0.003	−2.568	−0.012	−0.002
Constant	24.159 ***	1.193	20.255	21.819	26.498

Note: * p < 0.05 **: p < 0.01 ***: p < 0.001.

Finally, the study employed upward social comparison (X) as the independent variable, individual sports participation (W) as the moderating variable, and smartphone addiction (Y) as the dependent variable. The results are shown in Table 5. The moderating effect of individual sports participation on upward social comparison and smartphone addiction was not significant. (b = −0.005, p = 0.394).

Test of Moderating Effect of Individual Sports Participation (n = 925).

**Variables**	**Coefficient**	***SE***	***t***	**LLCI**	**ULCI**
Upward Social Comparison (X)	0.120 *	0.060	2.015	0.003	0.237
Sports Participation (W)	−0.223 ***	0.033	−6.758	−0.287	−0.158
X × W	−0.005	0.006	−0.853	−0.016	0.006
Constant	24..832 ***	1.379	18.002	22.125	27.539

Note: * $p < 0.05$ **: $p < 0.01$ ***: $p < 0.001$.
